# Fertility care interventions should be provided as the first line options for HIV+ serodiscordant couples who desire children in settings with affordable access to care, regardless of their fertility status

**DOI:** 10.7448/IAS.20.2.21294

**Published:** 2017-03-08

**Authors:** Lauren Zakarin Safier, Mark V Sauer

**Affiliations:** ^a^ Department of Obstetrics and Gynecology, New York Presbyterian-Columbia University Medical Center, Columbia University College of Physicians and Surgeons, New York, New York, USA

**Keywords:** HIV, human immunodeficiency virus, fertility, serodiscordant, assisted reproduction, sperm washing, *in vitro* fertilization, natural conception

## Abstract

**Introduction**: With increasing life expectancy, couples with at least one partner afflicted with HIV are more commonly pursuing the opportunity to have biologic offspring. Currently, there are no universally accepted recommendations regarding first line reproductive treatments for HIV serodiscordant couples lacking a history of infertility. We strongly believe that fertility care intervention should be the first line treatment, when affordably accessible, over natural conception for HIV serodiscordant couples to achieve pregnancy in a safe and efficacious manner.

**Discussion**: In the era of highly active anti-retroviral therapy, in combination with timed intercourse and pre-exposure prophylaxis for the HIV negative partner, some members of the medical community are arguing in favour of natural conception as a means of achieving pregnancy in this patient population. In our opinion, laboratory assisted fertility methods, including intrauterine insemination, *in vitro* fertilization, and intracytoplasmic sperm injection with semen washing should be the first line treatment recommendation for HIV serodiscordant couples desiring pregnancy for the following reasons: (1) abundance of evidence in the medical literature supporting the safety profile and efficacy of fertility care intervention in couples with HIV; paucity of data addressing safety of natural conception in comparison to fertility intervention techniques (2) unknown public health impact of promoting natural conception as a safe means of achieving pregnancy (3) ethical implications: patients should be offered the available and accessible treatment option posing the lowest possible known risk to the uninfected partner.

**Conclusions**: We believe that physician assisted fertility care, when affordably accessible, should be the treatment of choice over natural conception. While the preliminary data on natural conception in couples using highly active anti-retroviral therapy/pre-exposure prophylaxis/timed intercourse appears promising, we believe that this approach should be limited to patients in resource poor settings where more sophisticated measures do not exist or for patients that simply cannot afford subspecialty care. There are likely to be unknown psychological and behavioural factors impacted by promoting natural conception and diminishing the importance of safe sex practices. Additionally, it is our moral obligation to patients to offer the affordably accessible treatment interventions that pose the least known risk when considering reproductive options.

## Introduction

In the past, a diagnosis of HIV was associated with debilitating illness, a shortened life expectancy and untimely death. The advent of anti-retroviral therapy (ART) led to vast improvements in both quality of life and life expectancy of children and adults infected with the virus [1,2]. Currently, it is estimated that 37 million people worldwide are infected with HIV, and approximately 80% of these individuals are of reproductive age [3]. Improved prognosis has given patients hope for living normal lives and for long-term survival. Therefore, it is not surprising that today people living with HIV commonly pursue opportunities to have biologic offspring [4], but in ways that minimize known risk of transmission to their unaffected partner.

In 2012, the United States Food and Drug Administration (FDA) approved emptricitabine/tenofovir disoproxil fumarate as a pre-exposure prophylaxis (PrEP) measure to lessen HIV acquisition in sexually active adult men and women [5]. Published reports highlight the importance of ART and the potential for PrEP to reduce transmission of HIV in sexually active serodiscordant couples. The HIV Prevention Trial Network noted that utilizing ART in the infected partner in combination with PrEP in the HIV-seronegative partner, lowered HIV transmission by 96% [6]. Although preliminary, these measures appear to dramatically reduce risk to seronegative partners interested in conception.

Physician assisted reproductive interventions intended to aid individuals with HIV were met with resistance from the medical community, perhaps because of the presumed shortened lifespan of the HIV infected parent, or over concerns for the risk of horizontal and vertical transmission to the uninfected partner and child. Additionally, there were theoretical worries related to HIV infecting laboratory personnel who process gametes/embryos, as well as the potential for cross contamination of other patients’ embryos/gametes. Since 2001, the Centers for Disease Control and Prevention (CDC) in the United States encouraged health care professionals to provide information and support for HIV-infected couples who wish to explore their reproductive options, but without a treatment recommendation [7]. In 2015, the Ethics Committee of the American Society for Reproductive Medicine published an opinion stating that there is no longer reason to withhold fertility services from people living with HIV at clinics with necessary resources to provide care and that couples should be referred to providers offering risk-reducing therapies [8]. The World Health Organization similarly states that couples should be educated about the local infertility and prenatal services, the types of chemoprophylaxis available to reduce the risks of transmission to her child and, if in a serodiscordant relationship, HIV prevention approaches to minimize the risk of infection transmission to a partner when trying to conceive [9]. Reproductive assistance to HIV serodiscordant couples appears to allow conception while minimizing transmission risk. It is estimated that a female partner of an HIV-seropositive male has a 0.1 % risk of acquiring HIV following an act of unprotected intercourse [10]. The CDC estimates a risk of 4/10,000 per one act of vaginal intercourse, but sites factors that increase risk of HIV transmission including sexually transmitted diseases, acute and late stage HIV infection, high viral load, and number of exposures [11]. Although this risk is relatively low, it is certainly not negligible. Properly performed fertility care interventions seem to further lessen this risk by avoiding unprotected sex altogether. Reproductive methods such as sperm washing by density gradient ultracentrifugation and swim up techniques, followed by intrauterine insemination (IUI), *in* *vitro* fertilization (IVF), and/or intracytoplasmic sperm injection (ICSI) appear to be effective.

Currently, there are no universally accepted recommendations regarding first line reproductive treatments for HIV serodiscordant couples lacking a history of infertility. In developing countries, assisted reproductive technologies are not available, and therefore other methods of minimizing risk through natural conception are recommended. Strategies include delaying conception attempts until the partner living with HIV is on ART with suppressed viral loads, receiving treatment for other sexually transmitted infections (STIs), and limiting unprotected sex to times known to be associated with peak fertility potential. A strategy of managed conception in the setting of full viral suppression can minimize, yet likely does not fully eliminate, HIV transmission risk [12]. However, in the current era of ART and PrEP, pregnancy through natural conception is increasingly mentioned as an accepted transmission preventive strategy for HIV-serodiscordant couples, even in *developed* countries [13]. Yet, there remains a lack of sufficient evidence based results to assess the true safety of medical intervention versus natural conception, and promoting timed intercourse might produce an unintended negative psychological and behavioural result on serodiscordant couples if professional guidelines for safe sex are relaxed. We believe an ethical obligation exists to minimize the risk of transmission, and it seems prudent to continue to advise patients that medical interventions employing gamete preparation and laboratory based assisted reproduction should remain the preferred treatment option for serodiscordant couples who have access to such modern methods. In cases where such interventions are not possible due to access or affordability, other risk reducing methods should be discussed and employed.

## Discussion

Laboratory assisted fertility methods, including intrauterine insemination, *in vitro* fertilization and intracytoplasmic sperm injection in combination with semen washing have been advocated for over 25 years to decrease the risk of transmission. Regardless of the method chosen, controlling the disease of the partner living with HIV with ART is important to success. Appropriate ART to suppress viral replication and prevent progression to AIDS, is of vital importance in minimizing risk regardless of how conception is achieved.

For female HIV-seropositive individuals, non-coital ovulatory vaginal or intrauterine insemination is a low technology method offering a safer option to achieve pregnancy than sexual intercourse. There are limited data on the use of high-technology assisted reproductive methods for HIV-seropositive women, which include IUI, *in vitro* fertilization (IVF), and ICSI, although these procedures represent high cost interventions and access to care may be limited [13]. Additionally, maintaining a suppressed viral load during the course of pregnancy minimizes the risk of vertical transmission to the offspring. The risk of HIV in the newborn is estimated to be 13-30% in mothers not receiving preventive support and medication, whereas the use of ART during pregnancy and labour, combined with delivery by caesarean section, and avoidance of breast feeding are effective measures that reduce vertical transmission risk to less than 2% [9,14,15].

For HIV-seropositive males, risk reduction requires prolonged intervention given the possibility of passing virus through seminal fluid. The basic principle underlying assisted fertility methods for males living with HIV involves separating motile spermatozoa from seminal plasma and non-seminal cells that may harbour virus. The process of “sperm washing” in order to reduce transmission risk was introduced in 1989 by Semprini [16]. Sperm washing (SW) is achieved by combining density gradient centrifugation followed by sperm “swim-up”. The main location of the HIV inoculate in the male genital tract is within the seminal plasma (as free virions) or in association with non-spermatic cells (epithelial cells or lymphocytes that have receptors for the virus) also found within the ejaculate [17], so that utilizing a spermatozoa concentrate free of these contaminants provides safety from sexual transmission. The processed specimen is likely to be uninfected and can then be used for IUI, IVF, or ICSI [18].

There have been numerous studies reporting the efficacy of sperm washing (SW) in combination with IUI in terms of pregnancy rates, live birth rates, and HIV transmission incidence [19–21]. One study of 6000 cycles of SW followed by IUI, reported no seroconversions of female partners or births of HIV-infected children [22]. However, in the United States, the few centres offering fertility care have typically utilized IVF with ICSI when treating HIV discordant couples. Presently, the CDC continues to recommend against SW-IUI in HIV-infected men because of a reported seroconversion that occurred in a woman who had been artificially inseminated with semen from her HIV-infected husband nearly three decades ago [23].

IVF with ICSI was offered to HIV serodiscordant couples to circumvent concerns related to “insemination” techniques. Using ICSI, a single spermatozoon is injected directly into the oocyte, minimizing transmission risk to a cellular level [18]. Also, HIV-seropositive men commonly have abnormal semen analyses, and poor quality ejaculates often yield lower spermatozoa for insemination after semen processing, making IVF-ICSI an attractive treatment option. In one study including 420 consecutively performed cycles (355 fresh and 65 frozen cycles in 181 couples), the overall clinical pregnancy rate per embryo transfer was 45%; ongoing/delivered pregnancy rate per embryo transfer was 37%. No maternal or neonatal HIV infections or deaths occurred [24].

Despite the safety and efficacy of fertility care interventions, including semen washing with IVF/ICSI or IUI, there remains an issue of access, even for clients for whom these interventions are affordable. In one study that sought to investigate policy on patient access to services at assisted reproductive technology centres in the United States, only 7.1% of centres reported that they offer care to HIV+ women and 18.1% reported that they offer ART or IUI when the male was HIV seropositive [25].

For couples in resource limited areas without access to assisted reproductive technology, achieving pregnancy is limited to natural conception. Given the prevalence of HIV infection in resource poor settings, it is critical that sexually active couples be provided appropriate counselling and guidance in terms of the safest manner in which to achieve pregnancy. If available, ART is recommended for the HIV positive partner, and unprotected intercourse, or self-insemination, is timed to ovulation to minimize exposure of the unaffected partner. Additionally, if available, PrEP should be provided for the HIV-seronegative partner around the time of unprotected intercourse. With these non-invasive interventions requiring no advanced reproductive technology, transmission risk appears to be decreased.

Despite reduced HIV transmission rates for couples utilizing ART with PrEP, it is our belief that laboratory based interventions should remain the preferred option for those that have access for the following reasons:

1. Abundance of evidence in the medical literature supporting the safety profile and efficacy of fertility care intervention in couples with HIV; paucity of data addressing safety of natural conception in comparison to fertility intervention techniques

To date, most research has looked at the efficacy and safety of SW- IUI and IVF-ICSI as fertility treatments among HIV serodiscordant couples in which the male partner is HIV-infected. Substantial evidence points to the relative safety of these procedures, although some methodological limitations impede the evaluation and comparison of these studies [13, 26]. The safety profile and efficacy of semen washing for HIV serodiscordant couples was recently further validated using a meta-analysis by Zafer et al. In this study, no HIV transmission occurred in 11,585 cycles of assisted reproduction with the use of washed semen among 3994 women. Among the subset of HIV-infected men without plasma viral suppression at the time of semen washing, no HIV seroconversions occurred among 1023 women after 2863 cycles of fertility treatment with the use of washed semen. Studies that evaluated HIV transmission to infants born to serodiscordant couples reported no cases of vertical transmission. Overall, 56.3% of couples (2357/4184) achieved a clinical pregnancy with the use of washed semen [26].

For natural conception, data on safety profile is limited. This risk of infection from attempts at conception via unprotected intercourse depends on viral load, the presence of sexually transmitted infections, and the length and frequency of exposure [27]. The safety of natural conception will be difficult to prove due to the generally low seroconversion rate during unprotected intercourse [28]. While attainment of suppressed HIV replication in blood is associated with undetectable HIV-RNA in seminal plasma [21], some studies have shown that HIV-RNA may be amplified in semen, although undetectable in plasma [29]. Additionally, the exact risk of HIV transmission in an individual couple is difficult to predict, as HIV may be intermittently present in the male and female genital tract at variable concentrations, sometimes irrespective of ART or genital tract infections and has been detectable in seminal plasma of 6.6 % of men who are using ART for at least six months [30]. In order to gain more data to support the safety profile of natural conception, it would be useful for large studies to be conducted in resource poor settings reporting on the safety and efficacy of natural conception, where this is the only available method of conception.

In one recent analysis of 91 HIV-serodiscordant couples, (43 HIV-seropositive males and 48 HIV-seropositive females), there were 196 unprotected sexual exposures, resulting in 100 conceptions and 97 newborns. There were no cases of HIV seroconversion in uninfected sexual partners [31]. There are similar studies reporting on the outcomes of natural conception cycles, however they are limited in number and sample size. Natural conception may be an acceptable option in HIV-serodiscordant couples in resource limited settings if HIV-positive individuals have undetectable viral loads on ART, combined with HIV counselling, PrEP and timed intercourse.

To date, there are no randomized controlled trials comparing natural conception with PrEP to IUI with semen washing or IVF/ICSI to demonstrate feasibility, safety and effectiveness. Until there is more evidence, fertility care interventions by reproductive specialists in collaboration with infectious disease physicians overseeing patient health and well-being should remain the standard approach for couples seeking pregnancy in couples who have affordable access to these services.

- Unknown public health impact of promoting natural conception as a safe means of achieving pregnancy

Unprotected sex has not been the standard of practice recommended by most practitioners and researchers caring for HIV serodiscordant couples. A central concern is that compromising the “safe sex” message for the purpose of conception, even if only during a woman’s fertile window, might have deleterious effects on condom use and public health more broadly [32]. Risk compensation occurs when individuals modify behaviour in response to an altered perception of the probability of harm [33]. In one study using mathematical modelling to assess the effect of PrEP, it was found that while approximately 2.7 to 3.2 million new HIV infections could be averted in southern sub-Saharan Africa over 10 years by prescribing PrEP (having 90% effectiveness) to individuals at highest behavioural risk and by preventing sexual disinhibition, this benefit could be lost, by sexual disinhibition and by high PrEP discontinuation [34]. Therefore, there is concern that recommending natural conception as a means to achieve pregnancy may result in increased risky behaviour and unprotected intercourse in couples.

In one study examining fear and anxiety in HIV-serodiscordant couples interested in assisted reproductive technologies, at baseline both men and women displayed high state anxiety levels, but despite their anxiety, women were prepared to take risks to fulfil their desire for a child [35]. To our knowledge, no such study exists for couples contemplating natural conception.

The impact on promoting natural conception as the first line treatment in terms of consequences on risk taking behaviour and the effect on anxiety levels have yet to be determined.

- Ethical implications: patients should be offered available and accessible treatments that pose the lowest possible known risk to the uninfected partner

Many ethical issues are brought to bear in caring for seropositive couples desiring conception. Central issues include: the welfare of the child, prevention of harm, access to care and discrimination, acceptable levels of risk, and professional obligations of health care workers. Today, with the addition of ART and subsequent prolonged lifespans, HIV infection is now regarded as a chronic illness, and as such, medical interventions should not be categorically withheld when treating HIV-seropositive patients. The risk of infection needs to be evaluated against the benefits of parenthood, and given the progress of medical care, we believe it is ethically acceptable to attempt pregnancy so long as couples and doctors take all reasonable precautions to prevent transmission, and understanding that parents are prepared to take care of children regardless of resulting medical conditions [8].

Health care providers take a Hippocratic Oath at the beginning of their medical careers, and pledge to first “do no harm.” It is our moral obligation to provide our HIV-seropositive patients with the safest, evidence based treatment options. To date, fertility care interventions utilizing sperm washing techniques have been the most extensively studied treatment alternatives and appear both safe and efficacious. We are obliged to present the current options and recommendations, and counsel patients accordingly. In cases where fertility care interventions are accessible and affordable to the patient, this should be first line recommendation. Natural conception utilizing timed intercourse with PrEP should also be discussed as a risk reducing method of achieving pregnancy. Acknowledging that cost and accessibility of fertility care is an issue for many patients, natural conception may be the only pragmatic recommendation for indigent patients, especially in the developing world, and in carefully selected populations this may be the most appropriate option to protect the public health.

In resource poor settings, where inarguably the burden of HIV exists and there is limited access to fertility care inventions, any and all risk reducing strategies should be discussed and employed in this patient population. Even with PrEP alone, there is a 90% decrease in the rate of transmission amongst HIV-serodiscordant couples (CDC HIV Fact Sheet). Utilizing natural conception strategies with PrEP in an indigent population will likely decrease the risk of transmission currently and provide important information in terms of whether or not this option, in couples who are actively attempting pregnancy over a six months or extended time frame, is as safe as the presently recommended fertility care inventions.

## Conclusions

As health care providers, it is our responsibility to introduce harm-reduction methods and safer conception alternatives for patients with HIV. Given the current knowledge, we believe that physician assisted fertility care should be offered as treatment of choice over natural conception. While the preliminary data on natural conception in couples using ART/PrEP/timed intercourse appears promising, offering a relatively simple, safe and less expensive option, we believe that this approach should be limited to patients in resource poor settings where more sophisticated measures do not exist or for patients that simply cannot afford subspecialty care. Natural conception can and should be discussed with patients as a matter of true informed consent. However, it should not be recommended as the first line approach due to the paucity of data on safety in comparison to better studied and long employed fertility care interventions. Future studies need to be performed to better demonstrate the safety of natural conception. There are likely to be unknown psychological and behavioural factors impacted by promoting natural conception and diminishing the importance of safe sex practices. Finally, it is our moral obligation to patients to offer treatment interventions that pose the least known risk as the primary reproductive option.

## References

[1] Carlsson-Lalloo E, Rusner M, Mellgren Å, Berg M. Sexuality and reproduction in HIV-positive women: a meta-synthesis.. AIDS Patient Care STDS. 2016 21;30(2):56–17. 26741804 10.1089/apc.2015.0260 PMC4753620

[2] Mmeje O, Njoroge B, Cohen CR, Temmerman M, Vermund SH. van der Poel S Achieving pregnancy safely in hiv-affected individuals and couples: an important strategy to eliminate hiv transmission from mother-to-child and between sexual partners. J Acquir Immune Defic Syndr. 2015 121;70(4):e155–9. 26334740 10.1097/QAI.0000000000000814 PMC10767706

[3] Joint United Nations Programme on HIV/AIDS (UNAIDS). Fact sheet: 2014 statistics. Available from http://files.unaids.org/en/media/unaids/contentassets/documents/factsheet/2014/20140716_FactSheet_en.pdf 12349391

[4] Moragianni VA Why are we still, 20 years later, depriving human immunodeficiency virus-serodiscordant couples of equal access to fertility care?. Fertil Steril. 2014;102(2):352–53. 24931207 10.1016/j.fertnstert.2014.05.023

[5] US FDA. FDA approves first medication to reduce HIV risk cited 2012 716 Available from: http://www.fda.gov/forconsumers/consumerupdates/ucm311821.htm

[6] Cohen MS, Chen YQ, McCauley M, Gamble T, Hosseinipour MC, et al Prevention of HIV-1 infection with early antiretroviral therapy. N Engl J Med. 2011;365:493–505. 21767103 10.1056/NEJMoa1105243 PMC3200068

[7] Centers for Disease Control and Prevention. Revised guidelines for HIV counseling, testing, and referral. MMWR Recomm Rep. 2001;50(RR–19):1–57. 11718472

[8] ASRM Ethics Committee HIV and infertility treatment: a committee opinion. Fertil Steril. 2015 Jul;104(1):e1–e8. 10.1016/j.fertnstert.2015.04.004 25956374

[9] Wilcher R, Cates W Reproductive choices for women with HIV. Bull World Health Organ. 2009 Nov;87(11):833–39. 10.2471/BLT.08.059360 PMC2770278 20072768

[10] Boily MC, Baggaley RF, Wang L, et al Heterosexual risk of HIV-1 infection per sexual act: systematic review and meta-analysis of observational studies. Lancet Infect Dis. 2009 2;9(2):118–29. 19179227 10.1016/S1473-3099(09)70021-0 PMC4467783

[11] CDC high risk behaviors Centers for Disease Control and Prevention. HIV risk behaviors, estimated per-act probability of aquiring HIV from an infected source, by Exposure Act. Dec 2015.

[12] Matthews LT, Baeten JM, Celum C, Bangsberg DR Periconception pre-exposure prophylaxis to prevent HIV transmission: benefits, risks, and challenges to implementation. Aids. 2010 824;24(13):1975–82. 20679759 10.1097/QAD.0b013e32833bedeb PMC3773599

[13] Chadwick RJ, Mantell JE, Moodley J, Harries J, Zweigenthal V, Cooper D Safer conception interventions for HIV-affected couples: implications for resource-constrained settings. Top Antivir Med. 2011 11;19(4):148–55. 22156217 PMC6148878

[14] Townsend CL, Byrne L, Cortina-Borja M, Thorne C, De Ruiter A, Lyall H, et al Earlier initiation of ART and further decline in mother-to-child HIV transmission rates, 2000-2011. Aids. 2014;28(7):1049–57. 24566097 10.1097/QAD.0000000000000212

[15] Mandelbrot L, Tubiana R, Le Chenadec J, Dollfus C, Faye A, Pannier E, et al No perinatal HIV-1 transmission from women with effective antiretroviral therapy starting before conception. Clin Infect Dis. 2015 121;61(11):1715–25. 26197844 10.1093/cid/civ578

[16] Semprini AE, Levi-Setti P, Bozzo M, Ravizza M, Taglioretti A, Sulpizio P, et al Insemination of HIV-negative women witgh processed semes of HIV-positive partners. Lancet. 1992;340:1317–9. 1360037 10.1016/0140-6736(92)92495-2

[17] Houzet L, Matusali G, Dejucq-Rainsford N Origins of HIV-infected leukocytes and virions in semen. J Infect Dis. 2014 1215;210 Suppl 3:S622–30. 25414416 10.1093/infdis/jiu328

[18] Van Leeuwan E, Reeping S, Prins J, Van Derveen F Assisted reproductive technologies to establish pregnancies in couples with an HIV infected man.. Neth J Med. 2009 9;67(8):322–7. 19767658

[19] Semprini AE, Macaluso M, Hollander L, Vucetich A, Duerr A, Mor G, et al Safe conception for HIV-discordant couples: insemination with processed semen from the HIV-infected partner. Am J Obstet Gynecol. 2013 531;208(5):402–e1. 23395921 10.1016/j.ajog.2013.02.009

[20] Barnes A, Riche D, Mena L, Sison T, Barry L, Reddy R, et al Efficacy and safety of intrauterine insemination and assisted reproductive technology in populations serodiscordant for human immunodeficiency virus: a systematic review and meta-analysis. Fertil Steril. 2014 831;102(2):424–34. 24951364 10.1016/j.fertnstert.2014.05.001

[21] Bujan L, Hollander L, Coudert M, et al Safety and efficacy of sperm washing in HIV-1-serodiscordant couples where the male is infected: results from the European CREAThE network. Aids. 2007;21:1909–14. 17721098 10.1097/QAD.0b013e3282703879

[22] Savasi V, Mandia L, Laoreti A, Cetin I Reproductive assistance in HIV serodiscordant couples. Hum Reprod Update. 2013;19:136–50. 23146867 10.1093/humupd/dms046

[23] Center for Disease Control and Prevention. HIV-1 infection and artificial insemination with processed semen. Morb Mortal Wkly Rep. 1990;39:246–55. 2109169

[24] Sauer MV, Wang JG, Douglas NC, Nakhuda GS, Vardhana P, Jovanovic V, et al Providing fertility care to men seropositive for human immunodeficiency virus: reviewing 10 years of experience and 420 consecutive cycles of in vitro fertilization and intracytoplasmic sperm injection.. Fertil Steril. 2009 6;91(6):2455–60. 18555235 10.1016/j.fertnstert.2008.04.013

[25] Stern J, Cramer C, Garrod A, Green R Access to services at assisted reproductive technology clinics: a survey of policies and practices. Am J Obstet Gynecol. 2001;184:591–7. 11262458 10.1067/mob.2001.111793

[26] Zafer M, Horvath H, Mmeje O2, Van Der Poel S, Semprini AE, Rutherford G, et al Effectiveness of semen washing to prevent human immunodeficiency virus (HIV) transmission and assist pregnancy in HIV-discordant couples: a systematic review and meta-analysis.. Fertil Steril. 2016 3;105(3):645–55. 26688556 10.1016/j.fertnstert.2015.11.028 PMC4779710

[27] Bekker LG, Black V, Myer L, Rees H, Cooper D, Mall S, et al Guideline on safer conception in fertile HIV-infected individuals and couples. South Afr J HIV Med. 2011;12:31–44.

[28] Gray RH, Wawer MJ, Brookmeyer R, Sewankambo NK, Serwadda D, Wabwire-Mangen F, et al Probability of HIV-1 transmission per coital act in monogamous, heterosexual, HIV-1-discordant couples in Rakai, Uganda. Lancet. 2001;357:1149–53. 11323041 10.1016/S0140-6736(00)04331-2

[29] Bujan L, Pasquier C People living with HIV and procreation: 30 years of progress from prohibition to freedom?. Hum Reprod. 2016 51;31(5):918–25. 26975324 10.1093/humrep/dew036

[30] Lambert-Niclot S, Tubiana R, Beaudoux C, Lefebvre G, Caby F, Bonmarchand M, et al Detection of HIV-1 RNA in seminal plasma samples from treated patients with undetectable HIV-1 RNA in blood plasma on a 2002–2011 survey. Aids. 2012 515;26(8):971–5. 22382146 10.1097/QAD.0b013e328352ae09

[31] Sun L, Wang F, Liu A, Xin R, Zhu Y, Li J, et al Natural conception may be an acceptable option in hiv-serodiscordant couples in resource limited settings. Plos One. 2015 115;10(11):e0142085. 10.1371/journal.pone.0142085 PMC4634930 26540103

[32] Wilson DP, Law MG, Grulich AE, Cooper DA, Kaldor JM Relation between HIV viral load and infectiousness: a model-based analysis. Lancet. 2008;372:314–20. 18657710 10.1016/S0140-6736(08)61115-0

[33] Eaton LA, Kalichman S Risk compensation in HIV prevention: implications for vaccines, microbicides, and other biomedical HIV prevention technologies. Curr HIV/AIDS Rep. 2007 12;4(4):165–72. 18366947 10.1007/s11904-007-0024-7 PMC2937204

[34] Abbas UL, Anderson RM, Mellors JW, Wainberg M Potential impact of antiretroviral chemoprophylaxis on HIV-1 transmission in resource-limited settings. Plos One. 2007 919;2(9):e875. 17878928 10.1371/journal.pone.0000875 PMC1975470

[35] Van Leeuwen E, Visser M, Prinis JM, Nieuwkerk PT, Vander Veen F HIV couples’ anxiety and risk taking during ART. Fertil Steril. 2008;90:456–8. 17961558 10.1016/j.fertnstert.2007.06.086

